# [Retracted] MicroRNA‑34a expression affects breast cancer invasion *in vitro* and patient survival via downregulation of *E2F1* and *E2F3* expression 

**DOI:** 10.3892/or.2024.8841

**Published:** 2024-11-11

**Authors:** Rui Han, Jing Zhao, Lingeng Lu

Oncol Rep 43: 2062–2072, 2020; DOI: 10.3892/or.2020.7549

Following the publication of the above paper, it was drawn to the Editor's attention by a concerned reader that there appeared to be overlapping sections of data in the three Transwell invasion assay images shown in Fig. 4A, and in the 3D sphere formation assay data shown in Fig. 5, on p. 2068; moreover, certain of the data featured in one of the data panels in Fig. 4A also appeared in a subsequent publication by the same research group in the journal *Scientific Reports*.

The authors requested the publication of a corrigendum to acount for the errors made in assembling the data in Figs. 4 and 5; however, after careful consideration of the matter, the Editor of *Oncology Reports* has decided that this paper should be retracted from the Journal on account of the number of issues that were identified, and a general lack of confidence in the data. The authors were asked for an explanation to account for these concerns, but the Editorial Office did not receive a satisfactory reply. The Editor apologizes to the readership for any inconvenience caused.

